# The acute influence of amateur boxing on dynamic cerebral autoregulation and cerebrovascular reactivity to carbon dioxide

**DOI:** 10.1007/s00421-023-05324-y

**Published:** 2023-09-28

**Authors:** W. E. G. Wallis, Q. Al-Alem, H. Lorimer, O. J. Smail, G. K. R. Williams, B. Bond

**Affiliations:** Exeter Head Impacts, Brain Injury and Trauma (ExHIBIT) research group, Sport and Health Sciences, Baring Court, St Luke’s Campus, University of Exeter, Exeter, EX1 2LU UK

**Keywords:** Head impacts, Brain health, Boxing

## Abstract

**Purpose:**

The purpose of this study was to investigate the acute effect of head impacts, sustained over the course of three rounds of amateur boxing, on indices of cerebrovascular function.

**Methods:**

Eighteen university amateur boxers (six female) completed three experimental trials in a randomised order; (1) three rounds of boxing (BOX), (2) an equivalent bout of pad boxing (where no blows to the head were sustained; PAD), and (3) a time-matched seated control trial (CON). Indices of cerebrovascular function were determined immediately before and 45 min after each trial. Specifically, dynamic cerebral autoregulation (dCA) was determined by considering the relationship between changes in cerebral blood velocity and mean arterial pressure during 5 min of squat-stand manoeuvres at 0.05 and 0.10 Hz. Cerebrovascular reactivity was determined using serial breath holding and hyperventilation attempts.

**Results:**

Participants received an average of 40 ± 16 punches to the head during the BOX trial. Diastolic, mean and systolic dCA phase during squat stand manoeuvres at 0.05 Hz was lower after BOX compared to pre BOX (*P* ≤ 0.02, effect size (*d*) ≥ 0.74). No other alterations in dCA outcomes were observed at 0.05 or 0.10 Hz. The number of head impacts received during the BOX trial was associated with the change in systolic phase (*r = *0.50, *P* = 0.03). No differences in cerebrovascular reactivity to breath holding or hyperventilation were observed.

**Conclusions:**

A typical bout of amateur boxing (i.e., three rounds) can subtly alter cerebral pressure-flow dynamics, and the magnitude of this change may be related to head impact exposure.

## Introduction

Despite the pluripotent benefits of sports participation, there is concern that individuals engaged in contact sports may be at an elevated risk of neurodegenerative disease and dementia (Lehman et al. 2012; Mackay et al. 2019). It has recently been argued that the repeated exposure to sub-concussive head impacts might explain at least some of this risk (Russell et al. 2021; Nowinski et al. 2022). Given the devastating nature of such diseases, and that no curative treatment is currently available for dementia, identifying the underlying pathophysiological pathways associated with repetitive head impacts is recognised as a contemporary, public health priority (Livingston et al. 2020).

The brain blood flow response to alterations in blood pressure (cerebral autoregulation; CA) and carbon dioxide (cerebrovascular reactivity; CVR–CO_2_) reflect critical regulatory processes. A growing body of evidence indicates that these responses may be altered in contact sport athletes (Bailey et al. 2013; Wright et al. 2018a; Marley et al. 2021; Owens et al. 2021). Bailey et al. (2013), identified that professional boxers presented with altered CA response to a sit-to-stand transition and a thigh cuff occlusion challenge, and impaired CVR–CO_2_ to hypercapnia (5% CO_2_ inhalation) and hypocapnia (hyperventilation) compared to their age, sex and fitness-matched peers. Importantly, the impairment in CVR–CO_2_ observed was only associated with the volume and intensity of sparring during training (“sparring index”) rather than frequency of total knockouts experienced over the course of a career, which tallies with the prevailing concern regarding the accumulation of sub-concussive impacts.

It is possible that these chronic changes in CA and CVR–CO_2_ are driven by the repeated, acute alterations following exposure to head impacts (Huber et al. 2016). Therefore, it is important to understand whether the regulation of brain blood flow can be acutely altered by head impacts. Recent evidence demonstrates that heading in football may acutely alter dynamic CA (dCA) during repeated squat-stand transitions (Smirl et al. 2022) and other metrics of cerebral blood flow regulation (Smirl et al. 2020). However, our own work, which utilised fewer headers, did not identify any acute changes in dCA during repeated squat-stand manoeuvres, nor CVR–CO_2_ to repeated breath holding or hyperventilation challenges (Jack et al. 2022). Therefore, a dose–response relationship might exist regarding the number of subconcussive head impacts, and acute alterations in brain blood flow regulation. For example, changes in dCA metrics after a season of contact sports are associated with the frequency of head impact exposures (Wright et al. 2018a). However, few experimental data are available in this emerging field.

As the primary aim of boxing is to deliver repetitive blows to the head to score points, or win by knock-out, the sport offers a unique model to observe the effects of repetitive head impacts. The purpose of this study was to characterise any changes in dCA and CVR–CO_2_ following a typical amateur boxing match. A secondary aim was to consider whether any such changes were related to the acute cumulative head impact exposure. We hypothesised that acute alterations in dCA would be apparent post boxing, and that these changes would be positively associated with the number of head impacts experienced.

## Methods

### Participants

Following institutional ethical approval, 18 university amateur boxers (6 female; age 21 ± 1 y; mass 76.5 ± 14.6 kg; stature 1.74 ± 0.09 m; body mass index 25.0 ± 2.3 kg/m^2^) provided written informed consent to take part in the study. Using our unpublished pilot data, we calculated a priori that 17 participants would be needed to detect a time by trial interaction effect for dCA (phase—as determined by transfer function analysis) in a repeated, within-measures design. Participants had 3 ± 2 years of boxing experience, were active members of the competitive university squad, and took part in at least 3 rounds of sparring per week. The number of concussions previously sustained were as follows: 0 concussions *n = *13, 1 concussion *n = *3, 2 concussions *n = *1 and 3 concussions *n = *1. None of these concussions occurred within the 3 months prior to our study. All participants had passed a medical examination and were members of England Boxing (formally the Amateur Boxing Association of England). Exclusion criteria included any contraindications to exercise, history of drug abuse, smoking and the use of any medication which could influence blood pressure, heart rate, or vascular function.

### Experimental design

Participants were paired according to sex and mass, to prevent physical mismatches. Following a preliminary visit to the laboratory for familiarisation of all procedures and collection of descriptive data, each pair completed three experimental trials in a randomised order; boxing (BOX), pad-boxing (PAD), and a seated control trial (CON). Each visit occurred at the same time of day, and were separated by approximately 1 week. Participants were instructed to arrive at the laboratory having avoided vigorous exercise (Burma et al. 2020b) and caffeine (Peng et al. 2022) in the 24 and 4 h prior, respectively.

The BOX trial consisted of three separate rounds, each 3 min in duration, separated by 1 min of rest, as specified by the English amateur boxing rulebook (England Boxing 2022). Participants all wore regulation weight boxing gloves (12 oz). Each BOX session was completed at a boxing club, so that it could be overseen by a medically trained boxing coach and video recorded from two separate angles. Participants were driven (~ 10 min) to the club and back again by a member of the research team. The PAD trial was performed in the laboratory, and designed to replicate the exertion of a boxing session but without the exposure to head impacts. Participants completed the same format (three rounds) of punching hand pads held by a member of the boxing club. The CON trial consisted of time-matched seated rest (11 min) in the laboratory. Experimental outcomes were collected immediately before and 45 min after each trial, which provided sufficient time to re-instrument participants and ensure they were at rest prior to re-assessment of dCA outcomes.

### Estimation of head impacts

Two researchers reviewed the video footage and independently tallied the number of head impacts received by each boxer. The estimated total number of head impacts sustained across all bouts never differed by more than 5 punches between the two researchers (intraclass correlation coefficient = 0.998, *P* < 0.01). Thus, an average of these two values was taken for each BOX trial.

### Instrumentation

Blood velocity in the middle cerebral artery (MCAv) was measured using transcranial Doppler ultrasonography (DWL, Compumedics, Germany) to provide an estimation of cerebral blood flow (Willie et al. 2011; Ainslie and Hoiland 2014). A 2 MHz probe was positioned over the right trans-temporal window and secured with an adjustable headset (DiaMon, DWL, Germany). The position of the probe and insonation depth were noted and closely replicated within an individual across trials. The between-day coefficient of variation for resting MCAv (taken as the mean baseline value prior to the first BH attempt) was 7.6%. Beat-by-beat mean arterial pressure (MAP) was continuously measured by finger photoplethysmography (Finometer PRO, Netherlands) with correction to account for any height differences between the index finger and heart. R–R intervals were quantified using a 3 lead electrocardiogram. Partial pressure of end-tidal carbon dioxide (P_ET_CO_2_) was sampled via a mouthpiece and recorded using an online gas analyser (ML206; ADInstruments, USA). A nose clip was applied throughout to ensure inspiration and expiration was performed through the mouthpiece. MCAv, MAP, heart rate and P_ET_CO_2_ were sampled continuously at 200 Hz and integrated using an analogue-to-digital converter (Powerlab; model—8/30, ADInstruments, USA) linked with a laptop computer. All data were stored for later analysis using commercially accessible software (LabChart v8, ADInstruments, USA).

### Cerebral autoregulation

Repeated squat-stand manoeuvres (SSM) were used to assess dCA, in line with current recommendations (Smirl et al. 2015; Panerai et al. 2023). Participants underwent two separate 5-min SSM at 0.05 and 0.10 Hz, achieving a ~ 90° knee-bend with each squat. The order of the two SSM protocols were kept consistent within a participant, but counterbalanced between participants. Participants were given 2 min seated recovery between tests and were instructed to stand still for 1 min prior to initiation of the remaining SSM to allow recovery from initial orthostatic hypotension (Narayanan et al. 2001).

Metrics of dCA were determined for each SSM frequency and quantified using transfer function analysis (Panerai et al. 2023), using dedicated software (Elucimed, Ensemble-R, New Zealand) which incorporated R–R interval calibration. Output summaries of coherence, phase (radians), gain (cm/s/mmHg) and normalised gain (%/%) for 0.05 Hz and 0.10 Hz SSM were calculated at the point estimates of the SSM frequency, verified by visual inspection of blood pressure and MCA_V_ power spectrum densities (Smirl et al. 2015). Due to evidence that dCA may be differentially regulated across the cardiac cycle (Smirl et al. 2018), and to provide insight which might otherwise be missed (Burma et al. 2020b), alterations in dCA metrics were further scrutinised using the systolic and diastolic components of each cardiac cycle. There was no evidence of phase wrap-around at any point estimate. One participant was removed from 0.10 Hz SSM analysis due to a poor MAP signal acquisition which resulted in an abnormally low coherence value (< 0.70) for one visit.

### Cerebrovascular reactivity to carbon dioxide

CVR–CO_2_ during hypercapnia and hypocapnia was measured whilst seated using a repeated breath holding (BH) and hyperventilation (HV) protocol, respectively. This approach has been shown to be sensitive to alterations in CVR–CO_2_ in the days following concussion (Len et al. 2011). The BH and HV protocols were performed in a counterbalanced order between participants, but consistent within a participant. Baseline MCAv, MAP, cerebrovascular conductance (MCAv/MAP) and cerebrovascular resistance (MAP/MCAv) were measured for 1 min following approximately 5 min of seated rest. Following a normal inspiration, participants were then instructed to hold their breath for 20 s whilst remaining relaxed. Participants were coached to avoid performing the Valsalva manoeuvre during their first familiarisation visit. This was repeated five times in total, interspersed by 40 s recovery. The CVR–CO_2_ to repeated BH was quantified as the percentage increase in the greatest MCAv value above baseline following each breath hold attempt (BH_MCAv%_), in line with previous work from our laboratory (Koep et al. 2020, 2021; Jack et al. 2022). To better isolate the reactivity to carbon dioxide and minimise the influence of any changes in MAP or Valsalva manoeuvre on MCAv, individual breath hold attempts were removed from analysis if MAP increased by more than 15 mmHg. Nine such attempts (out of a total of 540) were rejected across the whole study in total. In addition, the percentage change in MCAv was expressed per unit change in P_ET_CO_2_ after each BH attempt (BH_CVR_).

A separate 1 min MCAv and PETCO2 baseline period was provided for the HV protocol. Participants then hyperventilated at 36 breaths per minute for 20 s, followed by 40 s of recovery. This was repeated five times in total. HV_MCAv%_ was determined as the percentage decrease in MCAv from baseline after each hyperventilation period. This percentage change in MCAv was also expressed per unit change in P_ET_CO_2_ after each HV attempt (HV_CVR_).

### Statistical analyses

Separate three (trial) by two (timepoint) repeated measures analysis of variance tests were used to determine changes in dCA metrics and resting haemodynamic outcomes, whilst three (trial) by two (timepoint) by five (attempt) analysis of variance assessments were used to identify any differences in CVR–CO_2_ outcomes. Fisher’s least significant differences post hoc analyses were used to explore any significant interaction effects. The main and interaction effects are presented using the *P* value and partial eta squared (*η*_p_^2^), which was interpreted as small (< 0.06), moderate (0.06 < *η*_p_^2^ < 0.14) and large (> 0.14). The magnitude of post-hoc pairwise comparisons were explored using standardised effect sizes (Cohen’s *d*) and interpreted as small (< 0.20), moderate (0.20–0.50) and large (> 0.50) (Cohen 1988). Pearson’s correlation was used to explore any relationship between the number and estimated accumulated force of received head impacts and the change in cerebrovascular outcomes. All statistical analyses were completed using SPSS (IBM Corp, USA, v26.0).

## Results

Each participant completed all trials in their entirety. Participants received an average of 40 ± 16 punches to the head during the BOX trial (range 22–68). The club medic did not note any potential concussive events, and no symptoms of concussion were reported by the participants.

### Resting haemodynamic data

No significant interaction effects were observed for baseline MCAv (*P* = 0.11, *η*^2^ = 0.12), MAP (*P* = 0.73, *η*^2^ = 0.02), P_ET_CO_2_ (*P* = 0.18, *η*^2^ = 0.11), cerebrovascular conductance index (*P* = 0.45, *η*^2^ = 0.05) or cerebrovascular resistance index (*P* = 0.92, *η*^2^ < 0.01).

### Squat stand manoeuvres at 0.05 Hz

**Fig. 1:**
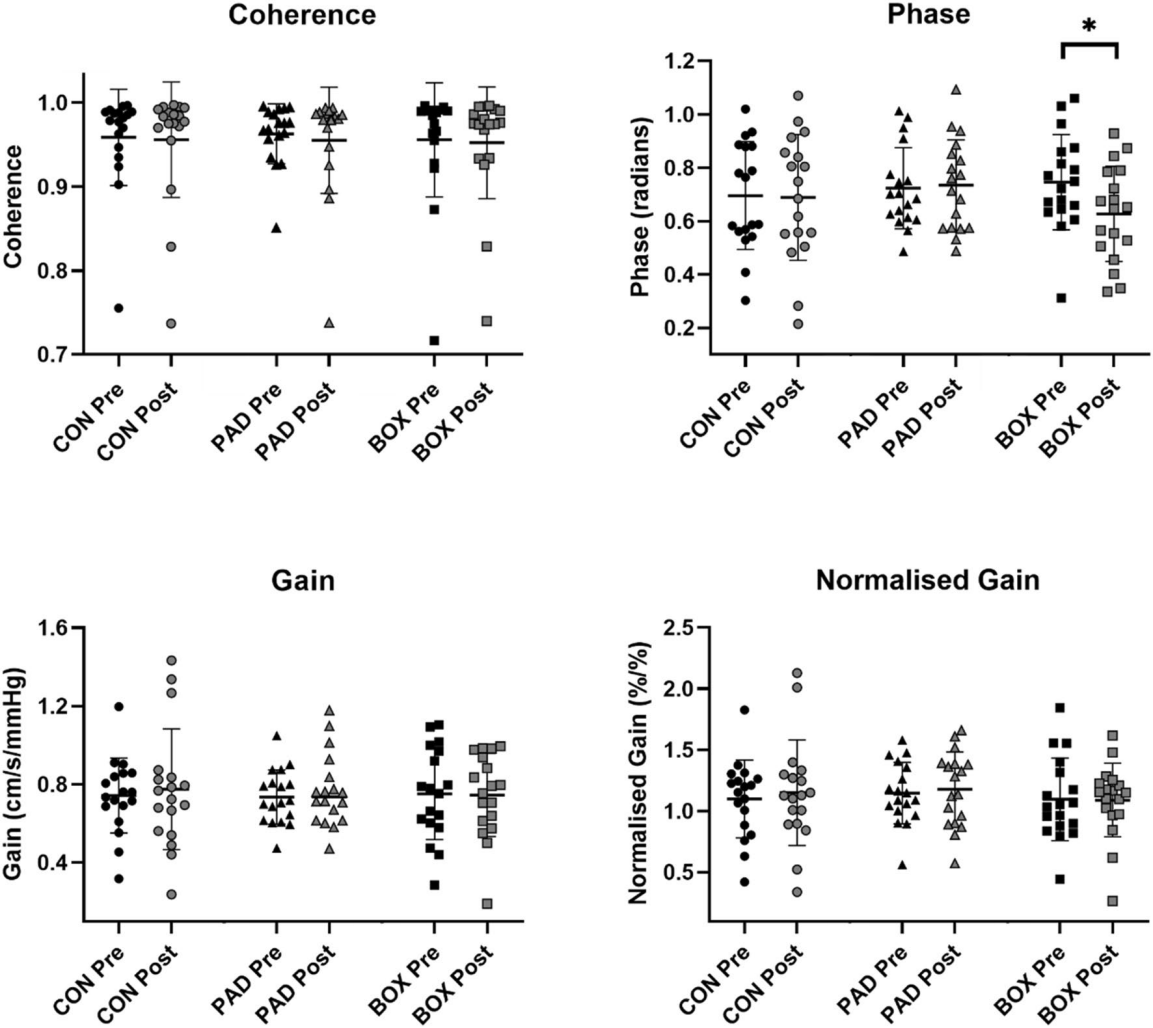
Transfer function analysis output from the 0.05 Hz frequency of the squat-stand manoeuvres across all three conditions (CO*N = *seated control trial, PAD = pad boxing (no head impacts) and BOX = boxing). Individual participant data are plotted along with the mean (horizontal line) and standard deviation (error bars). * Indicates a significantly reduced phase after the boxing trial when compared to pre-boxing measures (*P* = 0.02, *d* = 0.74)

There was no interaction effect for the power spectrum densities for MAP (*P* = 0.13, *η*^2^ = 0.11) or MCAv (*P* = 0.07, *η*^2^ = 0.16) during SSM at 0.05 Hz (Table 1). No trial by time interaction effects were observed for mean coherence (*P* = 0.98, *η*^2^ = 0.01), gain (*P* = 0.80, *η*^2^ = 0.01), or normalised gain (*P* = 0.78, *η*^2^ = 0.02) (Fig. 1). A lack of time by trial interaction was also observed for these dCA metrics during diastole (*P* > 0.24, *η*^2^ < 0.08 for all) and systole (*P* > 0.25, *η*^2^ < 0.08 for all). However, the trial by time interaction for mean phase was *P* = 0.05, *η*^2^ = 0.16. Post-hoc analysis of the interaction revealed a lower mean phase after BOX compared to pre-boxing (0.62 ± 0.17 vs 0.75 ± 0.18 radians, respectively, *P* = 0.02, *d* = 0.74). Phase was also significantly lowered after BOX in both systolic (1.00 ± 0.12 vs 1.33 ± 0.12 radians, *P* = 0.01, *d* = 2.75,) and diastolic (0.52 ± 0.05 vs 0.65 ± 0.04 radians, *P* = 0.01,* d* = 2.87) portions of the cardiac cycle.

**Table 1:** Power spectrum densities of oscillations in MAP and MCAv during SSM

	CON Pre	Post	PAD Pre	Post	BOX Pre	Post	*P* value
0.05 Hz squat-stand							
MAP PSD (mmHg)^2^/Hz	38,048 ± 21,410	31,897 ± 20,222	39,419 ± 23,944	35,970 ± 23,993	34,261 ± 16,861	39,020 ± 26,370	0.13
MCAv PSD (cm/s)^2^/Hz	26,568 ± 11,914	18,307 ± 11,914	24,075 ± 18,889	25,962 ± 30,277	24,048 ± 21,991	25,520 ± 19,335	0.19
0.10 Hz squat-stand							
MAP PSD (mmHg)^2^/Hz	29,986 ± 15,216	30,334 ± 11,538	22,588 ± 13,514	24,649 ± 8757	21,291 ± 6526	22,302 ± 10,342	0.91
MCAv PSD (cm/s)^2^/Hz	45,173 ± 30,852	39,380 ± 25,533	32,728 ± 18,595	41,951 ± 33,136	32,077 ± 20,734	34,360 ± 19,346	0.17

Data are displayed as mean ± standard deviation. The *P* value represents the ANOVA trial by time interaction

**Fig. 2:**
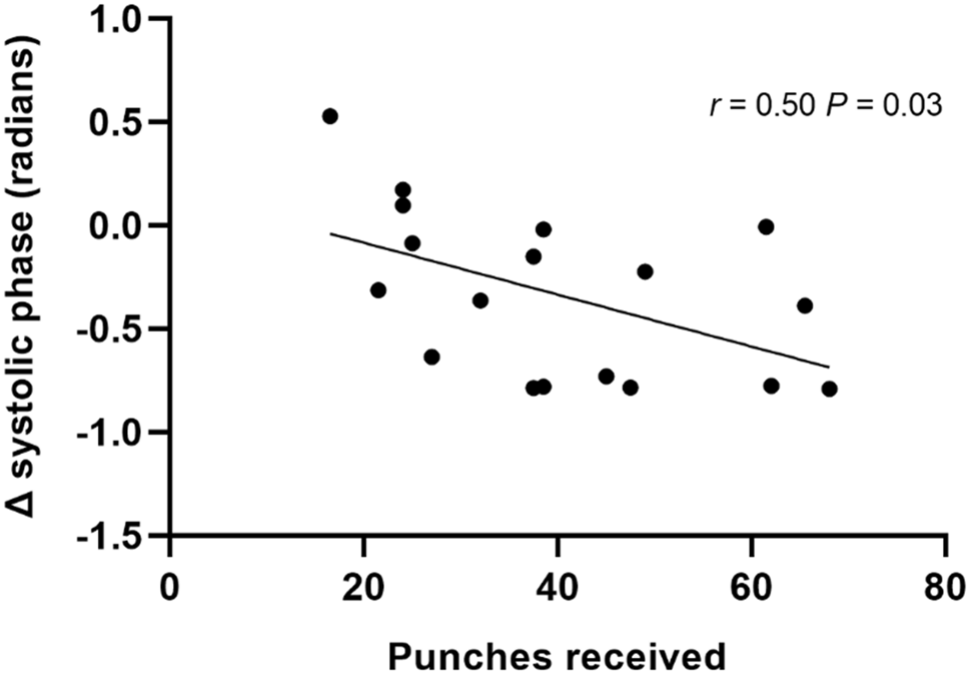
The association between the total number of head impacts and the post boxing change (Δ) in systolic phase during squat stand manoeuvres at 0.05 Hz

The change in systolic phase post-boxing (delta phase) was related to the number of head impacts received (*r = *0.50, *P* = 0.03; Fig. 2). This relationship was not observed for the change in diastolic or mean phase post boxing (*r < *0.18, *P* > 0.47). The change in phase across the cardiac cycle post-boxing was never significantly associated with years of boxing experience (*r < *0.36, *P* > 0.15).

### Squat stand manoeuvres at 0.10 Hz

**Fig. 3:**
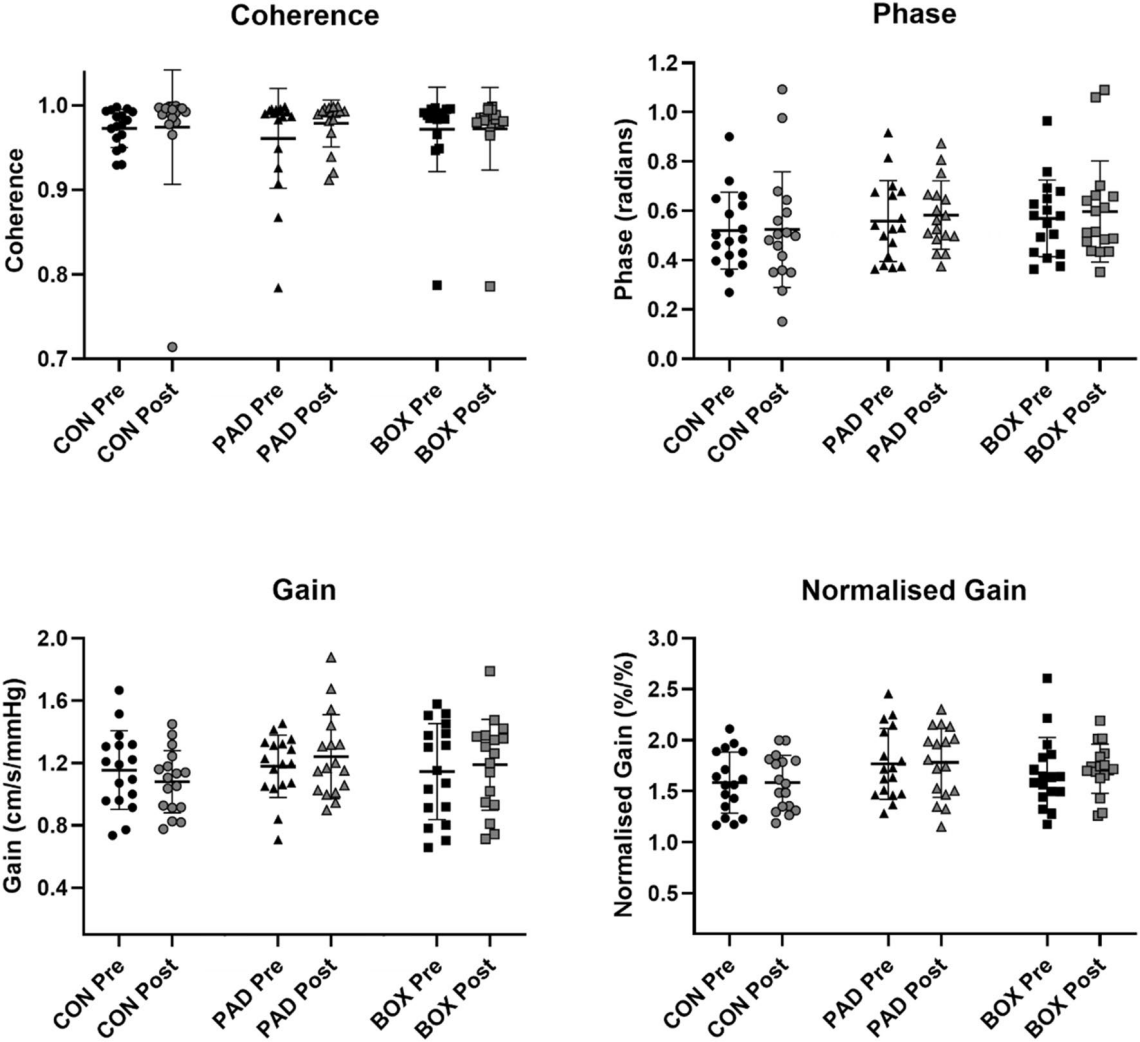
Transfer function analysis output from the 0.10 Hz frequency of the squat-stand manoeuvres across all three conditions (*CON* seated control trial, *PAD* pad boxing (no head impacts) and *BOX* boxing). Individual participant data are plotted along with the mean (horizontal line) and standard deviation (error bars). Repeated measures ANOVA revealed no significant interaction effect for any outcome (*P* > 0.26, *η*^2^ < 0.08 for all)

There was no time by trial interaction effect for MAP (*P* = 0.91, *η*^2^ = 0.01) or MCAv (*P* = 0.17, *η*^2^ = 0.11) power during SSM at 0.10 Hz. There was no significant interaction effect for mean coherence (*P* = 0.67 *η*^2^ = 0.02), phase (*P* = 0.90, *η*^2^ = 0.01), gain (*P* = 0.26, *η*^2^ = 0.08), or normalised gain (*P* = 0.89, *η*^2^ = 0.01; Fig. 3). Furthermore, no significant interaction effects in dCA metrics were observed in the diastolic (*P* > 0.10, *η*^2^ < 0.13 for all) or systolic (*P* > 0.13, *η*^2^ < 0.12 for all) portions of the cardiac cycle.

### Cerebrovascular reactivity to carbon dioxide

The breath holding protocol typically increased P_ET_CO_2_ by 3.1 ± 1 mmHg, which was never different (trial by time interaction *P* = 0.37, *η*^2^ = 0.08). The MCAv responses to breath holding was never altered in the study. Specifically, no significant interaction effects were observed for BH_MCAv%_ (*P* = 0.73, *η*^2^ = 0.05; Table 2), and this inference remained when normalising to P_ET_CO_2_ changes (BH_CVR_ *P* = 0.72, *η*^2^ = 0.03).

**Table 2:** Cerebrovascular reactivity to each breath hold challenge, expressed as the peak percentage change in middle cerebral artery blood velocity above baseline following each breath hold attempt

	CON (%)	PAD (%)	BOX (%)
Pre			
Attempt 1	40.3 ± 13.4	43.4 ± 17.9	38.7 ± 8.4
Attempt 2	46.9 ± 22.4	42.0 ± 12.2	33.2 ± 14.5
Attempt 3	43.5 ± 16.4	44.3 ± 19.9	37.2 ± 14.4
Attempt 4	37.6 ± 13.2	39.2 ± 14.4	38.7 ± 13.1
Attempt 5	40.8 ± 13.2	40.4 ± 15.6	34.9 ± 13.0
Mean	41.4 ± 15.6	40.8 ± 15.8	36.6 ± 12.7
Post			
Attempt 1	36.1 ± 14.6	34.3 ± 10.5	40.2 ± 15.6
Attempt 2	44.3 ± 17.6	36.5 ± 15.5	35.7 ± 9.0
Attempt 3	43.3 ± 19.3	39.8 ± 14.8	41.8 ± 15.3
Attempt 4	38.5 ± 17.8	34.5 ± 11.9	37.0 ± 12.9
Attempt 5	35.8 ± 17.3	33.2 ± 10.0	44.2 ± 15.7
Mean	39.0 ± 16.8	35.2 ± 13.4	39.6 ± 13.9

Data are presented as means ± standard deviations. Repeated measures ANOVA revealed no significant interaction effect (*P* = 0.73, *η*^2^ = 0.05)

*CON* control trial, *PAD* padwork trial, *BOX* boxing trial

Participants typically experienced reductions in P_ET_CO_2_ of 12.8 ± 2.3 mmHg after each hyperventilation attempt (trial by time interaction *P* = 0.16, *η*^2^ = 0.11). There was no interaction effect for HV_MCAv%_ (*P* = 0.20, *η*^2^ = 0.13; Table 3). No significant differences were observed when expressing this percentage change in MCAv per unit change in P_ET_CO_2_ (HV_CVR_ *P* = 0.24, *η*^2^ = 0.18).

**Table 3:** Cerebrovascular reactivity to each hyperventilation challenge, expressed as the percentage change in middle cerebral artery blood velocity from baseline following each 20 s hyperventilation

	CON (%)	PAD (%)	BOX (%)
Pre			
Attempt 1	− 32.0 ± 14.1	− 33.4 ± 11.7	− 36.1 ± 11.3
Attempt 2	− 25.7 ± 9.2	− 20.8 ± 25.9	− 30.6 ± 10.5
Attempt 3	− 25.6 ± 9.1	− 14.5 ± 33.3	− 26.6 ± 11.9
Attempt 4	− 18.0 ± 13.6	− 26.8 ± 12.1	− 25.3 ± 11.8
Attempt 5	− 23.5 ± 7.8	− 26.3 ± 7.7	− 23.6 ± 9.4
Mean	− 25.0 ± 15.0	− 24.4 ± 16.9	− 28.4 ± 11.6
Post			
Attempt 1	− 32.2 ± 14.8	− 30.1 ± 10.5	− 31.3 ± 12.3
Attempt 2	− 29.4 ± 10.1	− 33.4 ± 13.5	− 30.1 ± 12.0
Attempt 3	− 24.6 ± 15.6	− 28.7 ± 12.0	− 25.7 ± 10.8
Attempt 4	− 23.6 ± 9.9	− 28.4 ± 11.1	− 24.0 ± 15.1
Attempt 5	− 21.0 ± 16.0	− 27.2 ± 15.6	− 24.5 ± 13.7
Mean	− 26.2 ± 12.8	− 29.6 ± 12.5	− 27.1 ± 11.9

Data are means ± standard deviations. Repeated measures ANOVA revealed interaction effect (*P* = 0.20, *η*^2^ = 0.13)

## Discussion

The aim of this study was to identify whether a single bout of amateur boxing acutely alters putatively important metrics of brain blood flow regulation. We observed that many indices of dCA and CVR–CO_2_ were unchanged following three rounds of boxing; however, there was a delay in the ability to buffer the oscillations in MAP (i.e., phase) when performing SSM at the 0.05 Hz frequency. The absence of such changes in the PAD trial, and the observed association between the change in phase and the number of head impacts received, indicate that subconcussive blows to the head may acutely alter the cerebral pressure–perfusion relationship.

Alterations in the timing offset between changes in MAP and MCAv have previously been observed in male professional boxers (Bailey et al. 2013). Specifically, these authors identified a slower rate of MCAv regulation in boxers using the thigh cuff occlusion technique. Recent interventional work demonstrated that 40 headers, which is similar to the mean number of head impacts received in the present study, augmented (rather than attenuated) phase in young healthy footballers (Smirl et al. 2022). We are unable to reconcile this difference in the direction of the acute change in phase, although it is possible that the nature of the head impact exposure (a soccer heading drill, compared to 3 rounds of boxing) may have a contributory role. For example, greater rotational, but not linear, head accelerations have been reported in collegiate soccer and heading compared to university boxing (Doan et al. 2022), and the consequences of these stimuli are not equal (Zhang et al. 2006; Lota et al. 2022). The physiological challenge of three rounds of boxing (i.e., sympathetic arousal) is also likely to be distinctly different from a heading drill. A decrease in phase has been observed during SSM following a season of contact sports, and the magnitude of this change was also positively associated with the accumulation of head impact accelerations (Wright et al. 2018a). Interestingly, no changes in phase have been observed after 6 headers, despite replicating the study design and dCA methodology (Jack et al. 2022). The correlation between head impacts received and the change in dCA phase observed here suggests that a greater number of headers may have elicited such alterations. Taken together, it appears that alterations in dCA are possible in the acute aftermath of subconcussive head impacts.

The frequency-dependent nature of the subtle change in phase after BOX, where significant differences were observed during SSM at 0.05 Hz but not 0.10 Hz, may indicate alterations in myogenic rather than sympathetic mechanisms of regulation (Hamner et al. 2010; Hamner and Tan 2014). Such findings differ to the changes in phase only observed during 0.10 Hz SSM after concussion (Wright et al. 2018b) and subconcussive impacts (Wright et al. 2018a; Smirl et al. 2022). Collectively, this indicates that autoregulatory changes following head impacts might not be reserved to just one regulatory pathway. Similarly, we observed no changes in dCA gain, which aligns with the response post heading (Smirl et al. 2022). This indicates that perturbations in the speed of the buffering response may occur independently of changes in the ability to dampen the magnitude of the change in MCAv during MAP oscillations. We also report that phase was lower during diastole and systole, although only the latter correlated with the number of head impacts sustained. Differentiating phase in diastole and systole is important given that the oscillations during systole are understood to be more extensively buffered than their pressure-passive diastole counterpart, presumably to provide protection against haemorrhage (Smirl et al. 2018). Future research is needed to explore myogenic and sympathetic regulation mechanisms following head trauma, with acknowledgement of phase during diastole and systole. This work would also benefit from considering the time course of any such changes, as we cannot extrapolate our findings beyond the single measure obtained 45 min post BOX in our study.

The present study found no evidence of alteration in CVR–CO_2_ following three rounds of amateur boxing. Impairment in CVR has been observed in boxers (Bailey et al. 2013), footballers with a history of heading (Marley et al. 2021) and following a season of professional rugby (Owens et al. 2021). This suggests that a longer time frame of exposure is needed for alterations in CVR–CO_2_ to manifest; however, none of the aforementioned studies used breath-holding as their hypercapnic challenge. Whilst breath-holding has been advocated as a cerebrovascular stimulus (Tancredi and Hoge 2013; Pinto et al. 2020), our protocol did not provide the same magnitude of increase in P_ET_CO_2_ which would be expected with traditional CO_2_ breathing challenges (Koep et al. 2022), nor the same amount of control afforded by end-tidal forcing approaches (Howe et al. 2020; Carr et al. 2021). Such an approach was beyond the scope of this study, and open circuit challenges are not without their issues (Burley et al. 2020). It has been demonstrated that only ~ 65% of the MCAv response to a single breath-hold attempt is related to elevations in arterial CO_2_ (Przybyłowski et al. 2003). Therefore, our BH protocol is not the same CO_2_ stimulus as those used by others in this field (Bailey et al. 2013; Marley et al. 2021; Owens et al. 2021), which elicited greater increases in P_ET_CO_2_ (approximately 8–10 mmHg compared to 4–6 mmHg), and such comparisons are made with caution. However, we replicated the approach which has been shown to be acutely sensitive to concussion (Len et al. 2011). Furthermore, CVR–CO_2_ determined by breath-holding has been shown to be sensitive to sub-concussive head impacts sustained over the course of a season in female footballers (Svaldi et al. 2017). Finally, we observed no alterations in CVR–CO_2_ following hyperventilation, which did elicit more profound changes in P_ET_CO_2_ (average change − 12.8 ± 2.3 mmHg) that are consistent with other investigations (Len et al. 2011; Bailey et al. 2013; Owens et al. 2021). Therefore, these data indicate that alterations in CVR–CO_2_ might not be apparent immediately after three rounds of amateur boxing.

It is important to highlight that our study assessed the influence of three, 3 min rounds of boxing, which is the official length of a competitive British Universities and Colleges Sport amateur boxing bout and consistent with the current format of men’s Olympic boxing. However, a typical sparring session may include more rounds, and such training sessions may occur more than once in a single week. Therefore, understanding the time course in the recovery of dCA metrics would also be a valuable addition to our work. It has been shown that alterations in dCA following subconcussive head impacts may persist for 2 week post-season (Wright et al. 2018a), but no study has yet sought to precisely quantify this time course. This is contextually significant, as there is no standardised recovery time following a competitive bout at amateur level. This concern regarding such habitual exposure to head impacts, and the potentially unachievable length of time required for indices of cerebrovascular function to recover, has been raised elsewhere (Owens et al. 2021). Given that no untoward events or suspected concussions were reported by the club’s medical professional in this study, and that alterations in dCA can occur in the absence of traditional concussion-related symptoms (Wright et al. 2018b), it is important to understand whether head impacts or boxing during a period where dCA is already altered is associated with enhanced sensitivity to cerebrovascular change or heightened risk. To this end, further research should explore the effects of multiple sparring sessions across the week, and how this accumulates across a season, which may be more representative of amateur and professional boxing training.

The present study is the first controlled, experimental study to document metrics of brain blood flow regulation following amateur boxing. The within-measures design and inclusion of a boxing trial without head impacts (PAD) is a considerable methodological strength. However, there are some limitations in our design which should be acknowledged. First, the inclusion criteria outlined that participants only had to have at least 1 year boxing experience. Whilst our cohort are representative of university amateur boxers, this meant that participants were not standardised to skill or experience level. One result of this approach might be the high number of head impacts sustained in three rounds of boxing. It is possible that more skilled boxers may receive fewer blows to the head (Davis et al. 2015). However, it can also be argued that the force of those successful punches might be greater (Doan et al. 2022). These data, therefore, cannot be extrapolated to professional boxing, or other martial arts. Previous interventional studies in this field have attempted to standardise the head impacts—for example, launching a football at a designated speed (Jack et al. 2022; Smirl et al. 2022). In the absence of this control, the inclusion of instrumented mouth guards would be a valuable addition to better describe the nature of head impacts beyond the total number of blows to the head received (i.e., the magnitude and direction of accelerations). This would also help contextualise head impacts in boxing against other sports and provide some insight regarding any possible exposure threshold for changes in dCA. For example, recent data demonstrate that the linear and rotational accelerations of the head experienced during university boxing may be similar to collegiate American football (Doan et al. 2022).

Recent advances in this field have been made, whereby dCA can be separately scrutinised during periods of transient increases and decreases in MAP (Labrecque et al. 2021). This is insightful, as transient hypotension presents a more demanding autoregulatory challenge (Brassard et al. 2017; Labrecque et al. 2021). Unfortunately, it was not possible to consider this directionality in our data, so we cannot comment on whether the alterations in dCA observed are reserved only to periods of transient hypotension or hypertension, or prevail regardless of the direction of MAP change. Providing such insight would be valuable, and should be considered in future studies.

Another shortfall of this study is that it was unable to detect an effect of sex, as only 6 women were recruited. A sex effect on metrics of dCA during SSM is not a consistent finding (Labrecque et al. 2019a; Burma et al. 2020a), but may be particularly relevant in trained women (Labrecque et al. 2019b). Furthermore, women may experience greater brain microtrauma than men following sub-concussive head impacts of a similar magnitude (Rubin et al. 2018), possibly due to differences in neck strength and thus head movement (Caccese et al. 2018). In addition, we report here that the change in phase was not related to years of boxing experience, to broadly consider whether chronic exposure to head impacts might influence the acute response. It has been shown elsewhere that concussion history does not influence the recovery of dCA metrics in the weeks following a concussion in young healthy athletes (Wright et al. 2018b). However, our sample typically had only 3 years of prior boxing experience at amateur level, and far more work is needed to consider this research question. Whether or not a boxer with > 10 years of experience, who may well present with differences in dCA metrics at baseline (Bailey et al. 2013), might demonstrate a different response post boxing remains unknown.

TCD provides valuable temporal resolution when considering the reactivity to changes in arterial pressure and carbon dioxide concentrations. However, the assumption that MCAv is proportional to volumetric flow is contingent on an unchanging vessel diameter (Ainslie and Hoiland 2014). Typically this is accepted when P_ET_CO_2_ is within ~ 8 mmHg of eucapnia (Coverdale et al. 2014; Verbree et al. 2014). As is typical during SSM (Smirl et al. 2015, 2022; Burma et al. 2020a; Jack et al. 2022), this was achieved during our dCA assessment, but was exceeded during the hyperventilation protocol. However, our hyperventilation challenge replicated a protocol known to be sensitive to change in the days following a concussive event (Len et al. 2011), and we observed similar decreases in P_ET_CO_2_. Finally, a further shortcoming of our use of TCD is that we could not perform bilateral assessments of cerebral blood velocity due to technological restrictions. Thus, we were unable to quantify the haemodynamic responses beyond the conduit artery insonated (i.e., the MCA). Identifying whether acute head impacts alter brain blood flow regulation in the posterior cerebral circulation remains an interesting avenue for future research.

## Conclusion

Using a within-measures, controlled, experimental approach, our study demonstrates that subtle alterations in the cerebral perfusion–pressure relationship can occur in the aftermath of a typical amateur boxing bout. Specifically, dCA phase was lower (i.e., slower) during SSM at 0.05 Hz after 3 rounds of boxing. This change occurred in the absence of any suspected concussive events, and without changes in the cerebrovascular reactivity to carbon dioxide. Further work is now needed to understand the longevity of this perturbation, whether the athlete is more susceptible to further autoregulatory or cerebrovascular changes during this time, and the long term implications of habitual exposure to subconcussive head impacts.

## Data Availability

The data which support the findings of this study can be made available upon reasonable request.

## Abbreviations

BH: Breath holding
BH_CVR_: Percentage change in MCAv per unit change in P_ET_CO_2_ after each BH attempt
BH_MCAv%_: Percentage increase in MCAv above baseline following each BH attempt
BOX: Boxing trial
CA: Cerebral autoregulation
CON: Control trial
CVR: Cerebrovascular reactivity
dCA: Dynamic cerebral autoregulation
HV: Hyperventilation
HV_CVR_: Percentage change in MCAv per unit change in P_ET_CO_2_ after each HV attempt
HV_MCAv%_: Percentage increase in MCAv above baseline following each HV attempt
MAP: Mean arterial pressure
MCAv: Middle cerebral artery blood velocity
PAD: Pad-boxing trial
P_ET_CO_2_: Partial pressure of end tidal carbon dioxide
SSM: Squat-stand manoeuvres
